# Palmelloid Formation and Cell Aggregation Are Essential Mechanisms for High Light Tolerance in a Natural Strain of *Chlamydomonas reinhardtii*

**DOI:** 10.3390/ijms24098374

**Published:** 2023-05-06

**Authors:** Nittaya Suwannachuen, Kantinan Leetanasaksakul, Sittiruk Roytrakul, Narumon Phaonakrop, Siriwan Thaisakun, Peerapat Roongsattham, Chatchawan Jantasuriyarat, Nuttha Sanevas, Anchalee Sirikhachornkit

**Affiliations:** 1 Department of Genetics, Faculty of Science, Kasetsart University, Bangkok 10900, Thailand; 2 Center for Advanced Studies in Tropical Natural Resources, National Research University—Kasetsart University, Bangkok 10900, Thailand; 3 Functional Proteomics Technology Laboratory, National Center for Genetic Engineering and Biotechnology, National Science and Technology Development Agency, 113 Paholyothin Road, Klong 1, Klong Luang, Pathum Thani 12120, Thailand; 4 Department of Botany, Faculty of Science, Kasetsart University, Bangkok 10900, Thailand

**Keywords:** *Chlamydomonas reinhardtii*, natural variation, high light, palmelloid, aggregation, protein

## Abstract

Photosynthetic organisms, such as higher plants and algae, require light to survive. However, an excessive amount of light can be harmful due to the production of reactive oxygen species (ROS), which cause cell damage and, if it is not effectively regulated, cell death. The study of plants’ responses to light can aid in the development of methods to improve plants’ growth and productivity. Due to the multicellular nature of plants, there may be variations in the results based on plant age and tissue type. *Chlamydomonas reinhardtii*, a unicellular green alga, has also been used as a model organism to study photosynthesis and photoprotection. Nonetheless, the majority of the research has been conducted with strains that have been consistently utilized in laboratories and originated from the same source. Despite the availability of many field isolates of this species, very few studies have compared the light responses of field isolates. This study examined the responses of two field isolates of *Chlamydomonas* to high light stress. The light-tolerant strain, CC-4414, managed reactive oxygen species (ROS) slightly better than the sensitive strain, CC-2344, did. The proteomic data of cells subjected to high light revealed cellular modifications of the light-tolerant strain toward membrane proteins. The morphology of cells under light stress revealed that this strain utilized the formation of palmelloid structures and cell aggregation to shield cells from excessive light. As indicated by proteome data, morphological modifications occur simultaneously with the increase in protein degradation and autophagy. By protecting cells from stress, cells are able to continue to upregulate ROS management mechanisms and prevent cell death. This is the first report of palmelloid formation in *Chlamydomonas* under high light stress.

## 1. Introduction

Plants and algae are the primary producers that support life on earth via oxygenic photosynthesis. Despite its vital role in photosynthesis, light can be damaging to photosynthetic organisms. Even though photosynthetic organisms absorb light for growth, excessive light can harm cells. When light levels exceed the photosynthetic capability of plants, the excess energy generates reactive oxygen species (ROS). They include both free radicals, such as superoxide (O_2_^−^) anions and hydroxyl radicals (^•^OH), and non-radicals, such as hydrogen peroxide (H_2_O_2_), and singlet oxygen (^1^O_2_). An excessive accumulation of ROS within cells generates oxidative stress, which destroys cellular components, such as proteins, lipids, and nucleic acids, hence impairing cell development and viability [1]. Thus, photosynthetic organisms have evolved a variety of acclimation responses to maintain a balance between the energy absorbed and the levels of ROS produced to maximize growth and minimize cell damage due to high light conditions.

Strategies to manage the excess light energy and the amount of ROS are categorized as short-term and long-term responses [2]. Short-term photoacclimatory reactions occur within seconds or hours. These reactions include changes in the redox state of the plastoquinone pool, cyclic electron movement around a PSI to alleviate a portion of the excessive pressure on the PSII, and the nonphotochemical quenching of excited chlorophyll states (NPQ) of excess excitation energy as heat, which includes energy dissipation via the interconversion of carotenoids of the xanthophyll cycle, and state transitions, in which the antenna system is transferred between photosystems [2,3,4]. In a period spanning hours to days, the long-term response includes changes in transcriptional and translational regulation, enzyme activation, and protein synthesis, and changes in protein stoichiometry [2,5]. These changes lead to the downregulation of the light-harvesting capacity of the organism, the upregulation of the ROS scavenging system by the accumulation of antioxidants, such as carotenoids and tocopherol (vitamin E), and antioxidant enzyme activities, such as superoxide dismutase (SOD), catalase, and peroxidases. Together, these mechanisms are known to be important for survival under light stress.

Many plant studies have contributed to our understanding of pathways involved in the high light response. The problem with plants, however, is that light is not spread uniformly throughout the organism. Differential reactions of diverse tissue types, many of which are not engaged in photosynthesis, further complicate the interpretation of data. Thus, measurements and estimations of numerous parameters will not reflect the values in photosynthetic cells. The unicellular green alga, *Chlamydomonas reinhardtii,* has been used as a model organism to study photosynthesis and growth under a variety of conditions, including light stress [6,7,8,9,10,11]. This alga is an excellent model organism for studying stress responses because it possesses a similar light-harvesting complex to those of higher plants. Many stress-responsive genes are present in its genome. It is unicellular, which makes it easy to analyze the physiological changes that occur within the cell in response to stress. Cells can be grown in a liquid culture with agitation to achieve the uniformity of light stress. Furthermore, the advancement of systems biology is made possible by the availability of genomic sequences [12].

Several studies have utilized *Chlamydomonas* as a model organism for evaluating high light responses [13,14,15,16]. Nevertheless, many essential mechanisms and proteins for adaptation to extreme light exposure remain unknown. More importantly, the vast majority of studies have been conducted on laboratory strains or high light tolerant mutants derived from laboratory strains [17,18]. The majority of laboratory strains came from a soil sample from Amherst, Massachusetts, taken by Gilbert Smith in 1945 and distributed among several laboratories [19]. As determined by whole-genome sequencing and extensive investigations on numerous laboratory strains and field isolates, *Chlamydomonas* is exceedingly polymorphic and has the greatest nucleotide diversity ranking among the eukaryotes [17,20]. Despite the availability of numerous field isolates with multiple phenotypic variations, these field isolates have never been used to dissect the pathways related to high light responses. Differences in phenotypes within the same species or natural variations can contribute to our understanding of the relative importance of various pathways. It can be used to identify the function of particular genes. In this study, two natural *Chlamydomonas* strains with opposing growth phenotypes under high light were investigated. The physiological responses and proteome modifications of a naturally high-light-tolerant strain were examined. The results indicate that this field isolate uses palmelloid formation and cell aggregation as a strategy for surviving severe light stress. The role of related proteins and the adaptation mechanism to high light stress are discussed.

## 2. Results

### 2.1. Two Natural Chlamydomonas Strains Displayed Different Growth Variations under High Light

**Figure 1:**
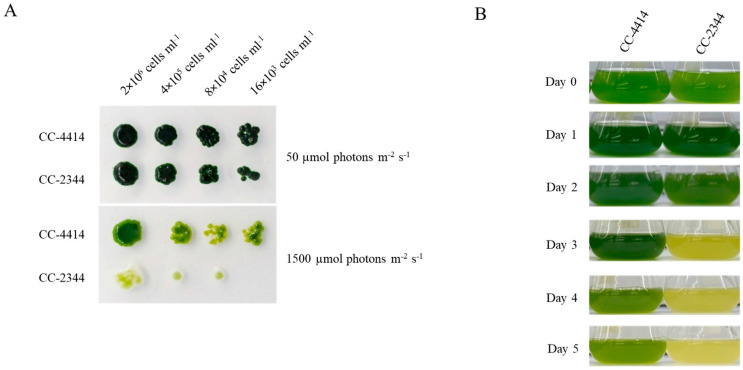
Growth of two *Chlamydomonas* strains exposed to high light stress. (**A**) Cells were diluted to a density of 2 × 10^6^ cells mL^−1^ and subsequently subjected to 5-fold serial dilutions. Cells were then spotted on TAP medium plates and cultivated under 50 and 1500 µmol photons m^−2^ s^−1^. Photos were taken after one week. (**B**) Cells were diluted to a density of 2 × 10^6^ cells mL^−1^ in liquid TAP medium and exposed to high light of 1500 µmol photons m^−2^ s^−1^ over a period of 5 days.

**Figure 2:**
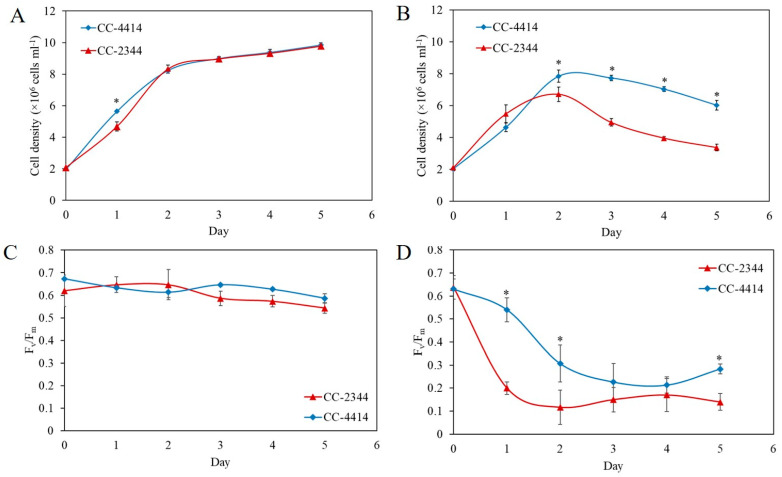
Growth and maximum quantum efficiency of PSII (F_v_/F_m_) of *Chlamydomonas.* Cells were grown under (**A**) low light at 50 µmol photons m^−2^ s^−1^ and (**B**) high light at 1500 µmol photons m^−2^ s^−1^. The cell density of each strain was assessed to compare the growth of the two strains over a period of 5 days. The F_v_/F_m_ values of the high-light-tolerant strain (CC-4414) and the high-light-sensitive stain (CC-2344) were measured under low light (**C**) and high light (**D**). All data are means ± SD (*n* = 3). Significant differences between the two strains on the same day are indicated with asterisks (*) (*p* < 0.05).

Daytime light levels in natural environments can alter the growth and development of cells. Various *Chlamydomonas* strains may react differently to different light intensities. Twenty *Chlamydomonas* strains, consisting of thirteen field isolates and seven laboratory strains, were examined on plates and in a liquid culture at varying light intensities to examine growth variations under high light. A few strains showed consistent resistance to high light after multiple rounds of testing, while a few strains demonstrated consistent sensitivity. Subsequently, two field isolates were chosen for further characterization. The strain CC-2344 was chosen as a sensitive strain based on it being one of the stains exhibiting the lowest F_v_/F_m_ value in high light and an early bleaching phenotype on the plate. This strain was isolated from Ralston, Pennsylvania. The selected high-light-tolerant strain, CC-4414, was chosen based on its interesting origin. According to the Chlamydomonas Resource Center website, this strain was isolated at 13,000 feet above sea level in Colorado (https://www.chlamycollection.org/, accessed on 21 May 2022), indicating that this strain has adapted to a hard environment, including high light intensity. In order to compare growth phenotype on plates, log-phase cells of the specified density were spotted onto plates and cultured under low light and high light. Under low light at 50 µmol photons m^−2^ s^−1^, these strains developed similarly, but under high light at 1500 µmol photons m^−2^ s^−1^, CC-2344 exhibited reduced growth and the bleaching of cell spots (Figure 1A). Similarly, when liquid cultures were shifted from low light to high light, CC-4414 began to turn pale green on day 4, while CC-2344 bleached and turned yellow on day 3. (Figure 1B). Variations in cell density were also seen in relation to growth. Under low light, both strains grew similarly over a period of 5 days (Figure 2A). On day 2, however, significant variations in cell density were detected under high light (Figure 2B). Even though both strains showed a reduction in cell density from day 2 to day 5 under high light, CC-2344 displayed a much lower cell density (Figure 2B).

### 2.2. Variation in Photosynthetic Efficiency and Pigment Levels

**Figure 3:**
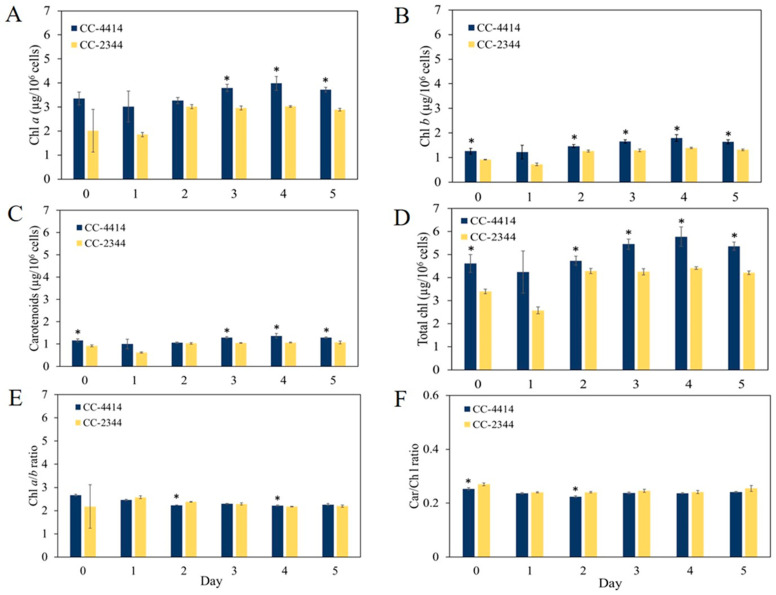
Pigment contents and composition of *Chlamydomonas* under low light. Photosynthetic pigments were measured from cells grown under high light at 1500 µmol photons m^−2^ s^−1^ and reported as (**A**) chlorophyll *a*, (**B**) chlorophyll *b*, (**C**) carotenoids, (**D**) total chlorophyll, (**E**) chlorophyll *a/b* ratio, and (**F**) carotenoid/chlorophyll ratio. All data are means ± SD (*n* = 3). Significant differences between the two strains on the same day are indicated with asterisks (*) (*p* < 0.05).

**Figure 4:**
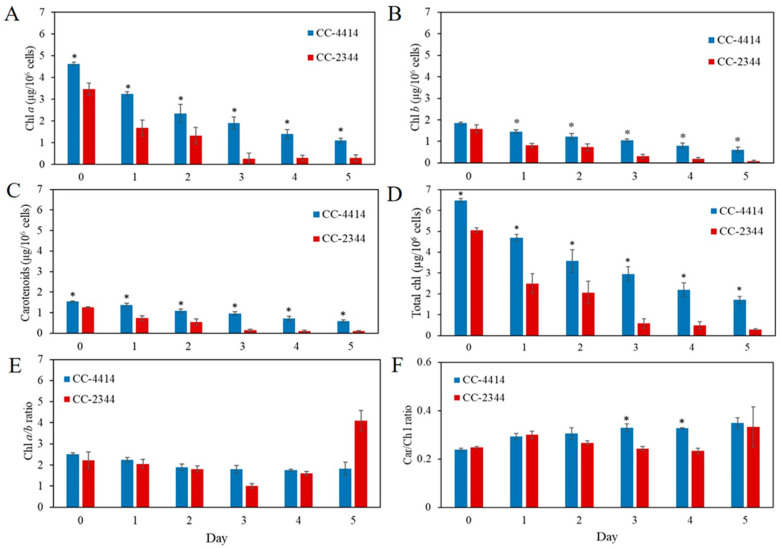
Pigment contents and composition of *Chlamydomonas* under high light treatment. Photosynthetic pigments were measured from cells grown under high light at 1500 µmol photons m^−2^ s^−1^ and reported as (**A**) chlorophyll *a*, (**B**) chlorophyll *b*, (**C**) carotenoids, (**D**) total chlorophyll, (**E**) chlorophyll *a/b* ratio, and (**F**) carotenoid/chlorophyll ratio. All data are means ± SD (*n* = 3). Significant differences between the two strains on the same day are indicated with asterisks (*) (*p* < 0.05).

Under low light conditions of 50 µmol photons m^−2^ s^−1^, the F_v_/F_m_ values of the two *Chlamydomonas* strains maintained a similar trend (Figure 2C). Following 24 h of exposure to high light intensity at 1500 µmol photons m^−2^ s^−1^, the F_v_/F_m_ value of CC-2344 dropped sharply to around 0.2, but the value of CC-4414 remained between 0.5 and 0.6 (Figure 2D). The values of CC-2344 declined slightly on day 2 and remained stable until the end of the study. In contrast, the value of CC-4414 continued to decrease from day 2 through day 4, before showing a slight recovery on day 5. At every time point, the CC-4414 value was greater than the CC-2344 value was. In response to light intensity, the levels and composition of photosynthetic pigments, primarily chlorophylls and carotenoids, fluctuated. To explore variations in the level and composition of photosynthetic pigments, the levels of chlorophylls and carotenoids were evaluated. Under low light, the levels of these pigments in CC-2344 were slightly lower than those in CC-4414 (Figure 3A–D). In response to a shift to high light intensity, chlorophyll *a*, chlorophyll *b*, total chlorophyll, and carotenoids decreased in both strains, while CC-4414 had significantly higher levels of all photosynthetic pigments than CC-2344 did (Figure 4A–D). The ratio of chlorophyll *a* to chlorophyll *b* remained unchanged in CC-4414 over five days, whereas the ratio significantly increased in CC-2344 on the last day (Figure 4E). CC-4414 had a significantly greater carotenoid/chlorophyll ratio beginning on day 3, whereas the ratio of CC-2344 remained unchanged (Figure 4F).

### 2.3. Responses to Oxidative Stress and Strategies to Mitigate Oxidative Stress

**Figure 5:**
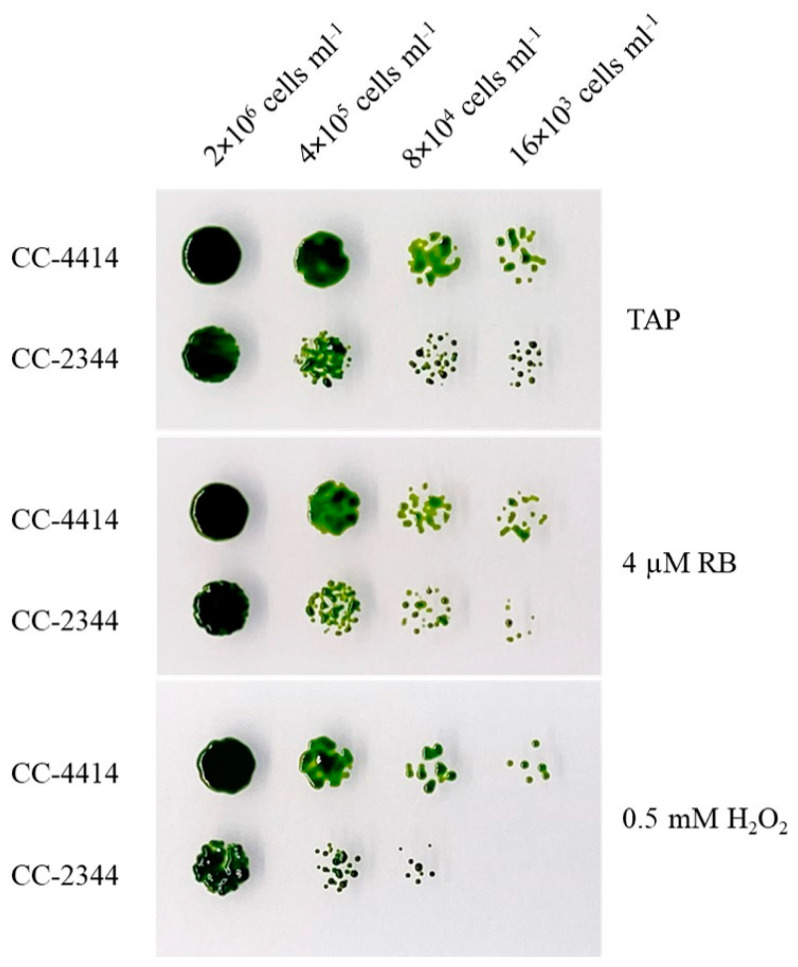
Growth of *Chlamydomonas* under oxidative stress. Cells were diluted to a density of 2 × 10^6^ cells mL^−1^ and subjected to 5-fold serial dilutions. Cells were spotted on TAP, TAP with Rose Bengal (RB), and TAP with hydrogen peroxide (H_2_O_2_). Plates were incubated under 50 µmol photons m^−2^ s^−1^, and photos were taken after 5 days.

To test if the differences in photosynthetic damage and pigment levels caused by high light were primarily attributable to cellular oxidative stress, oxidative stress was directly administered to plates using Rose Bengal (RB), which promotes the generation of singlet oxygen under light, and H_2_O_2_. Cells at the indicated cell density were spotted on plates containing these compounds. CC-2344 grew slightly slower than CC-4414 did under control conditions (Figure 5, top). Both RB and H_2_O_2_ inhibited the growth of both strains (Figure 5, middle and bottom, respectively). However, CC-4414 only slightly outperformed CC-2344 under these conditions.

**Figure 6:**
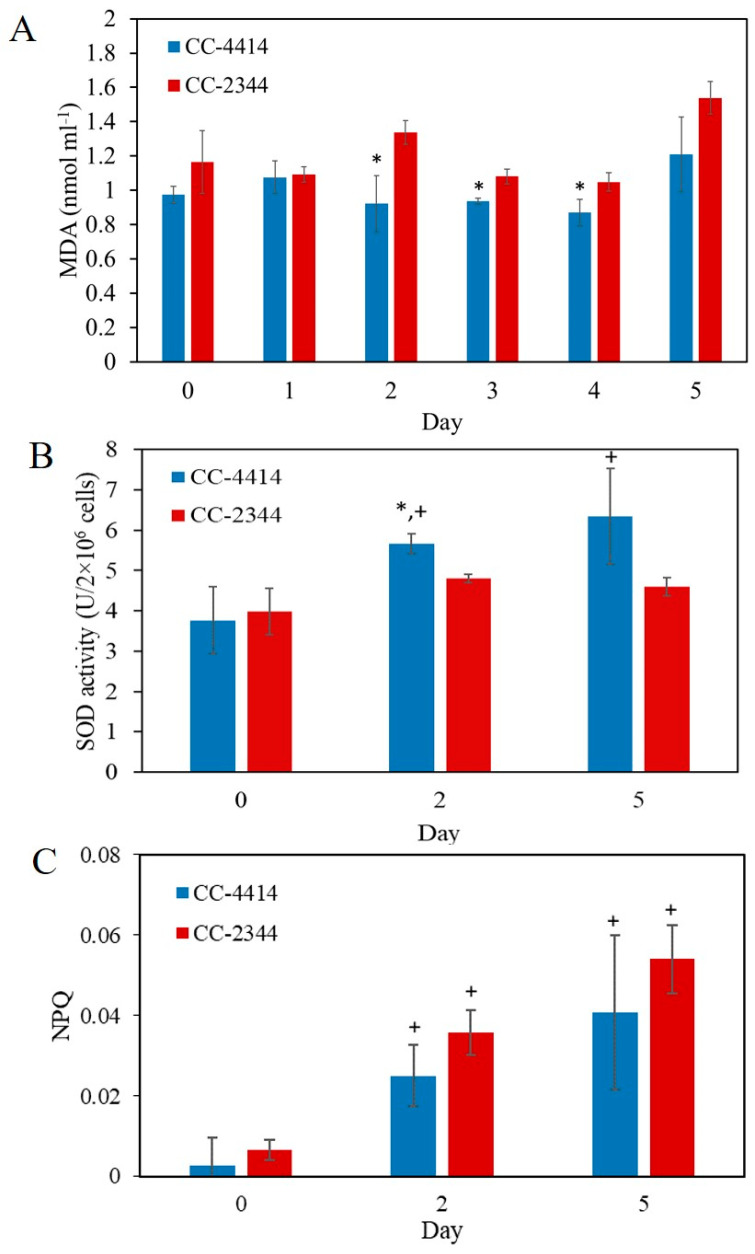
*Chlamydomonas* responses under oxidative stress. Cells were grown under high light at 1500 µmol photons m^−2^ s^−1^. (**A**) Lipid peroxidation was measured by the thiobarbituric acid reacting substance (TBARS) method. Oxidative stress was evaluated by detecting malondialdehyde (MDA) levels in cells. (**B**) The activity of superoxide dismutase (SOD) was measured to evaluate ROS scavenging activities. (**C**) Non-photochemical quenching (NPQ) of *Chlamydomonas*. All data are means ± SD (*n* = 3). Significant differences between the two strains are indicated with asterisks (*), while significant differences between each day and day 0 within the same strain are indicated with plus signs (+) (*p* < 0.05).

Biomolecules, including lipids, proteins, and nucleic acids, can be damaged by oxidative stress. Measuring the degree of lipid peroxidation is one approach for understanding cell damage. The thiobarbituric acid reactive substance (TBARS) method, which is used to analyze malondialdehyde (MDA) levels, can be used to assess the final products of polyunsaturated fatty acid peroxidation in the cells. This measurement provides an estimate of oxidative-stress-induced cell damage. Between days 2 and 4, the level of cell damage was lower in CC-4414 than it was in CC-2344 (Figure 6A). Under high light, the levels of MDA in CC-2344 increased slightly on days 2 and 5. Additionally, the MDA levels of CC-4414 under high light exposure were substantially lower than those of CC-2344 from day 2 to day 4 (Figure 6A).

Superoxide dismutase is one of the most important antioxidant enzymes in the defense against ROS-induced oxidative damage. CC-4414 exhibited a considerably higher level of SOD activity than CC-2344 did on day 2 when it was exposed to 1500 µmol photons m^−2^ s^−1^ of high light (Figure 6B). In addition, CC-4414 displayed a considerable increase in the SOD activity level on days 2 and 5 relative to day 0 after high light transfer. On day 2, higher SOD activity correlated with reduced MDA levels of CC-4414, showing that the detoxification mechanism was employed more. However, the distinction between CC-4414 and CC-2344 was relatively small.

NPQ can be used as an indicator of photoprotection mechanisms against excessive light stress. Both strains displayed a considerable increase in NPQ on days 2 and 5 following exposure to high light compared to that on day 0 (Figure 6C). However, no significant changes between the two strains were observed.

### 2.4. Proteome Analysis

**Figure 7:**
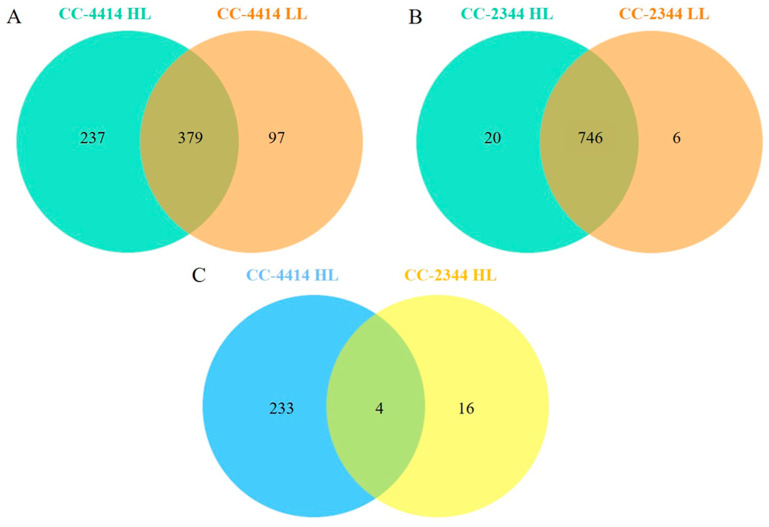
Venn diagrams showing proteins differentially expressed under low light of 50 µmol photons m^−2^ s^−1^ and two days after exposure to high light of 1500 µmol photons m^−2^ s^−1^. Comparison between low light and high light for CC-4414 (**A**). Comparison between low light and high light for CC-2344 (**B**). Comparison of proteins exclusively expressed in high light of CC-4414 (237 proteins) and CC-2344 (20 proteins) (**C**).

To further explore mechanisms that confer high light tolerance in CC-4414, we analyzed peptide sequences and identified differentially expressed proteins between the two strains under high light using liquid chromatography–tandem mass spectrometry (LC/MS-MS). Uniprot (http://www.uniprot.ort/, accessed on 8 March 2023) was used to search the peptide sequences database. A total of 379 proteins were shared between low light and high light treatments applied to CC-4414 (Figure 7A). Two hundred and thirty-seven proteins were specifically expressed in high light, and ninety-seven proteins were uniquely expressed in low light. In contrast, CC-2344 contained 746 proteins that were found in common under both high- and low light, which was nearly twice as many as those found in CC-4414 (Figure 7B). Merely 20 and 6 proteins of CC-2344 were distinct under high- and low light conditions, respectively. A Venn Diagram was constructed based on the proteins uniquely expressed in high light in each strain (Figure 7C). This Venn Diagram reveals that there were 233 proteins identified exclusively in CC-4414.

**Figure 8:**
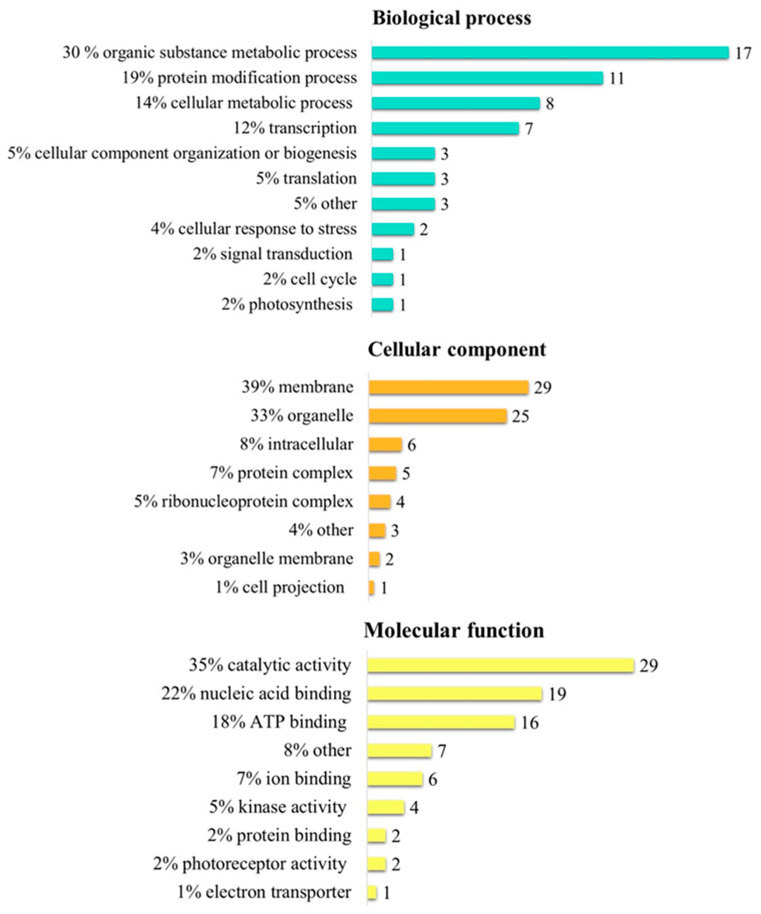
Gene Ontology (GO) enrichment analysis of proteins exclusively expressed in CC-4414 exposed to high light of 1500 µmol photons m^−2^ s^−1^ for 2 days. The GO terms are categorized as a biological process (**top**), cellular component (**middle**), or molecular function (**bottom**). The number of proteins for each GO term is indicated at the end of each bar graph.

To examine the adaptation and acclimation processes involved in the high light-tolerant phenotype, we focused on 233 proteins detected exclusively in CC-4414 under high light. They were categorized as various Gene Ontology (GO) processes. Under the biological process category, proteins were identified in the organic substance metabolic process (30%), the protein modification process (19%), the cellular metabolic process (14%), transcription (12%), cellular component organization or biogenesis (5%), translation (5%), the cellular response to stress (4%), photosynthesis (2%), signal transduction (2%), the cell cycle (2%), and others (5%) (Figure 8, top). Under the category of cellular component, the majority of expressed proteins were associated with the membrane (39%), organelle (33%), intracellular (8%), protein complex (7%), ribonucleoprotein complex (5%), organelle membrane (3%), cell projection (1%), and others (4%) (Figure 8, middle). According to their molecular function, the majority of proteins were involved in catalytic activity (35%), nucleic acid binding (22%), ion binding (7%), kinase activity (5%), protein binding (2%), photoreceptor activity (2%), electron transporter (1%), and others (8%) (Figure 8, bottom). 

In a detailed examination of these proteins under organic substance and metabolic process, two proteins related to oligosaccharide biosynthetic and metabolic processes, as well as proteins involved in DNA replication and transcription, were found (Table 1 and Table S1). Many protein kinases used for protein modification were also identified. Under the category of cellular component organization or biogenesis, an expansin was found. The bulk of the proteins in the category of cellular components are engaged in the cell membrane. Other than proteins involved in DNA and RNA structure and functions, such as helicase, histone demethylase, RNA ligase, and acetyltransferase, there were proteins involved in ubiquitination and glycosylation, including glycosyltransferase activity and glucosidase activity in the endoplasmic reticulum (ER). A detailed summary of all proteins with known GO terms is available in the Supplementary Materials (Tables S1–S3). Under high light, four proteins shared by CC-4414 and CC-2344 were identified. Table 2 lists an adenylate kinase, a potassium: proton antiporter, a copper homeostasis-related protein, and an unknown protein. Sixteen proteins were expressed exclusively in CC-2344 under high light. The functions of half of these proteins are unknown. The remaining proteins are related to membrane proteins or have nucleic acid-related activities (Table 3).

**Figure 9:**
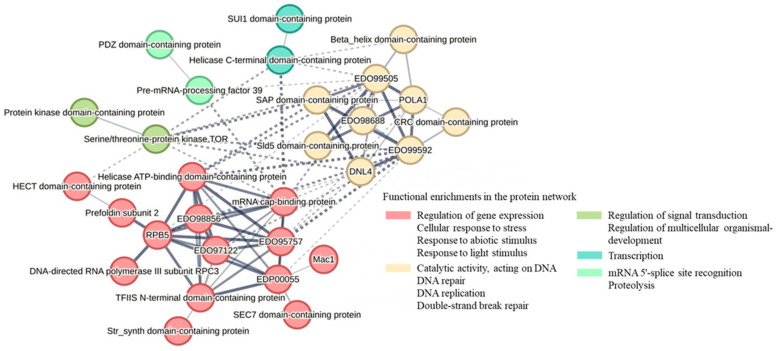
Predicted interaction network of significant proteins exclusively found in *Chlamydomonas* CC-4414 under high light treatment. STRING software was used to obtain a network of protein interactions.

The interactions among proteins found specifically in CC-4414 under high light were then determined using the STRING 11.5 database (http://string-db.org/, accessed on 25 February 2023). The presence of a protein group involved in the regulation of gene expression and the cellular response to abiotic stress, such as helicase ATP-binding domain-containing protein, HECT domain-containing protein, prefoldin subunit 2, SEC7 domain-containing protein, TFIIS N-terminal domain-containing protein, Mac1, mRNA cap-binding, Str synth domain-containing protein, Mac1, and mRNA cap-binding, is indicative of the regulation of gene expression (Figure 9). They were identified as ones that interact with proteins, such as protein kinase and the serine/threonine protein kinase TOR, which regulate signal transmission and multicellular organism development. The serine/threonine protein kinase TOR is physically and functionally conserved in eukaryotes and responds to environmental signals by regulating metabolic activities, growth, and survival. Additional proteins in the interaction network included DNA replication, DNA repair, and transcription proteins. In reaction to stress, Sld5 domain-containing protein and transcription initiation factor TFIIH subunit 2 affect DNA replication and transcription, respectively. Together, these predicted proteins may improve the tolerance to high light stress.

### 2.5. Cell Morphology and Aggregation

**Figure 10:**
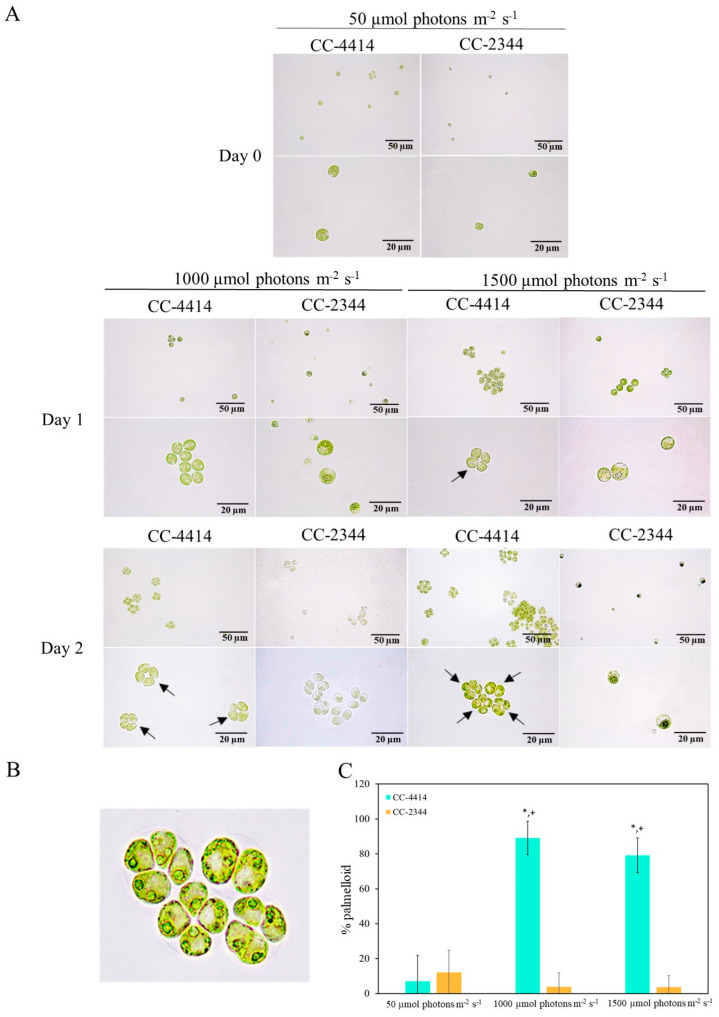
Morphological changes in *Chlamydomonas* were monitored via microscopy. (**A**) *Chlamydomonas* cells were cultured in TAP medium and placed under low light of 50 µmol photons m^−2^ s^−1^ and high light of 1000 and 1500 µmol photons m^−2^ s^−1^ for 2 days. Black arrows indicate examples of palmelloid structures in CC-4414. (**B**) A close-up image of palmelloid cells. (**C**) Percentage of palmelloid cells. Significant differences between the two strains are indicated with asterisks (*), while significant differences between each high light intensity and low light within the same strain are indicated with plus signs (+) (*p* < 0.05).

To examine the effects of light-level stress on the morphology of *Chlamydomonas*, cells were cultured in TAP media, and then subjected to high light. Under controlled low light conditions, both strains were single cells with a diameter of 10 µm and a cup-shaped chloroplast (Figure 10A). Cell aggregation and the development of palmelloid in CC-4414 occurred as early as day 1 under 1000 µmol photons m^−2^ s^−1^ and 1500 µmol photons m^−2^ s^−1^ (Figure 10A,B). Approximately, 80–90% of CC-4414 cells were in a palmelloid state (Figure 10C). In contrast, CC-2344 did not display cell aggregation or the formation of this structure to the same extent. Under high light, CC-2344 was identified as having damaged single cells with an enlarged round form and a diameter of around 15 µm, as well as smaller chloroplasts with a pale green color. 

## 3. Discussion

### 3.1. ROS Management

Environmental stresses impair photosynthesis and promote the production of ROS. The first line of defense is designed to prevent oxidative stress by decreasing ROS generation and increasing ROS elimination via multiple strategies, including non-photochemical quenching, alternative electron transport pathways, and the activation of antioxidant enzymes and pathways [2,21].

According to our findings, changes in F_v_/F_m_ preceded differences in cell density. F_v_/F_m_ is a sensitive indicator of photooxidative stress and measures the efficiency of photosynthesis. The tolerant strain appeared to recover more quickly in F_v_/F_m_, suggesting that CC-4414 underwent less cell stress due to photodamage than CC-2344 did. Lipid peroxidation was only different from day 2 to day 4, and the differences were not especially notable, aside from the oxidative damage to PSII. Chemicals that cause oxidative stress did not exhibit substantial differences among phenotypes. These findings suggest that the variations in growth could not be largely attributable to the ability to manage ROS levels. 

Long-term responses to high light are typically described as changing the size of the PSII antenna and decreasing the chlorophyll (Chl) *a*/*b* ratio [22,23]. The number of reaction center complexes, the PSI/PSII ratio, and/or the size of the LHC antennas are some examples of these structural changes [2]. According to earlier research, within six hours of exposure to high light, cells drop their amount of chlorophyll per cell by half compared to those grown in low light [7,24,25]. The Chl *a* content dropping could mean that PSII and PSI reaction centers are deteriorating [26]. By day 5 in our studies, both strains lost more than half of their total chlorophyll content. Under high light in cells that are undergoing exponential growth, the Chl *a*/*b* ratio either remains stable [7] or increases [27]. Our findings demonstrated that Chl *a*/*b* ratios of both strains were constant. High light causes *Chlamydomonas* to produce less chlorophyll per cell and produce more carotenoids per cell [28,29]. These mechanisms increase the neutralization of reactive oxygen species by carotenoids, while decreasing the input excitation of the PSII. According to our findings, this ratio in CC-4414 was very marginally increased, and the ratios of two strains did not differ until days three and four. As a result, raising the carotenoid content or car/chl ratio was not the primary strategy for CC-4414 to tolerate high light levels.

Measurements of NPQ and SOD activity were used to evaluate the capacity to manage ROS levels. Common responses for high light shift involve a decrease in light absorption and an increase in defensive mechanisms to counter photooxidative damage. In both strains, an early defense mechanism such as NPQ was similarly activated [2,4]. According to our findings, NPQ was one of the primary mechanisms used because both strains upregulate this pathway. However, NPQ did not significantly differ between the two strains. As such, NPQ was not the primary factor in the ability of CC-4414 to tolerate high light. The level of SOD activity was slightly higher in CC-4414. Consequently, controlling oxidative stress does play a role in its tolerance phenotype. The phenotypic changes in oxidative stress on plates, however, was not particularly striking. Thus, other factors must play a more significant role in light tolerance. In terms of proteome alterations, none of the proteins directly linked to the first line of defense were observed. This indicates that there was minimal variation between the two strains with regard to this first line of defense.

### 3.2. Protein Degradation by the Ubiquitin–Proteasome Pathway and Autophagy

The amount of light that is present throughout the day in a natural setting can reach 2000 µmol photons m^−2^ s^−1^. At 1500 µmol photons m^−2^ s^−1^, the high light intensity used in our work was quite high. Since the first line of defense between the two strains did not differ much, changes in other pathways must be involved. Autophagy and protein degradation are two processes that comprise the second line of defense [30].

Autophagy is a pro-survival self-degradative process in charge of removing any damaged or unneeded components, including proteins and membranes, and recycling them into necessary components under stress [31,32,33]. In *Chlamydomonas*, stress is strongly associated with autophagy induction. Examples of these stresses include nitrogen or carbon starvation, endoplasmic reticulum stress, carotenoid deficiency, and the impairment of starch biosynthesis [34,35,36,37]. ROS play a significant role in the regulation of autophagy in plants and algae [38]. This might be one of the reasons why the first line of defense in the high-light-tolerant strain, CC-4414, did not show a much better ability to regulate ROS than the sensitive strain did. The explanation could be that ROS need to be appropriately controlled in order to be at a level appropriate for their signaling functions. When the first line of defense is insufficient, many cellular systems, including autophagy, need to be activated by ROS.

The primary proteolytic mechanism for regulating protein degradation is the ubiquitin–proteasome system (UPS) [39,40]. The proteasome, a multiprotein complex, targets ubiquitinated proteins for destruction. In *Chlamydomonas*, stresses such as arsenate, copper, metal nanoparticles, cold, and chloroplast damage were shown to activate the UPS [41,42,43,44,45]. In the degradation of certain transcription factors, the UPS plays a significant role in plant stress responses [46,47].

In our protein–protein interaction network, serine/threonine protein kinase TOR, an ATG protein that monitors and controls metabolic processes, growth, and survival in response to environmental inputs, was identified (Figure 9). Through interacting with abiotic stimuli response proteins, including helicase ATP-binding domain-containing protein, mRNA cap-binding protein, and SAP domain-containing protein, this protein may play a significant role in the pro-survival mechanism of the high- light-tolerant phenotype. Several ubiquitin-related enzymes were also identified, including E3 ubiquitin-protein ligase, RBR-type E3 ubiquitin transferase, and ubiquitin carboxyl-terminal hydrolase. These are UPS members induced under high light in CC-4414.

### 3.3. Palmelloid Formation and Cell Clustering under Severe Stress

The UPS and autophagy are activated under moderate stress conditions, most likely as a result of ROS signaling, in order to remove toxic chemicals and recycle nutrients that enhance survival until optimal conditions are reached. However, under extreme stress conditions, alternative mechanisms are necessary to permit cell survival. Palmelloid production and cell aggregation have been suggested as stress tolerance mechanisms. These cellular structural changes may occur together with the UPS and autophagy [21].

Palmelloids consist of between four and sixteen cells enclosed by a cell wall and are the product of serial divisions driven by stress, without cell wall disintegration. Palmelloid formation has been observed under abiotic stresses, including the presence of predators, organic acids, EDTA, calcium deficiency, phosphorous deficiency, cadmium, salt, and acidic pH [48,49,50,51,52,53,54,55,56,57,58]. Many cell wall layers of these palmelloids indicated that the cells continue to divide within the mother cell, i.e., they are unable to separate and remain encased in a single membrane. Numerous proteins were discovered in the palmelloid secretome in response to salt stress during palmelloid development [54]. Cell wall proteins were involved in the generation and maintenance of palmelloids. Five cell wall proteins were upregulated. Expansin, a cell wall protein involved in cell enlargement, was the most significant one, followed by pherophorins-C5, C6, and C9 and a hydroxyproline-rich glycoprotein. Matrix metalloproteinase MMP3, cell wall glycoprotein GP3, pherophorins- C15 and -C17, and hydroxyproline-rich cell wall protein ISG were exclusively upregulated during the de-stress treatment, indicating probable alterations in cell wall composition. 

Cell aggregation can confer a protective role by shielding internal cells from stress, which is similar to biofilms and biological soil crusts [59,60]. In the case of cell aggregation, a recent study revealed that *Chlamydomonas* socializer (saz) mutants, which spontaneously form massive aggregations, are more resistant to abiotic stresses [30]. These aggregates differ in structure from palmelloids. The medium from the mutants could cause wild-type cells to aggregate. The analysis of the secretome showed several proteins associated with cell wall structure. Interestingly, the medium contained a sugar-rich extracellular matrix (ECM). Transcriptomic data revealed the upregulation of genes encoding pherophorins, proteases, lipases, and ECM-associated proteins, which may be involved in cellular communication, a change in their ability to interact, and the formation of a sugar-rich ECM.

Similarly, our proteome data revealed alterations in nucleic acid metabolism and protein modification, which led to alterations in membrane proteins as a major component of the cell. An expansin-like EG45 domain-containing protein was identified in CC-4414 following exposure to high light. This protein is likely involved in morphogenesis and other developmental events, such as cell wall breakdown [61]. An endoprotease, a calpain catalytic domain-containing protein belonging to the MEROPS peptidase family C2, was also present. This protein is an intracellular protease engaged in numerous crucial physiological functions that are regulated by calcium and has been shown to be potentially implicated in cell wall organization [62]. In addition, we identified a PDZ domain-containing protein with serine-type peptidase activity and the agonist-dependent activation of cell surface receptors [63]. Additionally, an SprT-like domain-containing protein involved in metalloendopeptidase activity and capable of activation by these endoproteases was identified. 

Under high light, the palmelloid structure of CC-4414 was easily noticeable. Some degree of aggregation was observed, although not to the same extent as that in *saz* mutants, where massive aggregation was found [30]. A small percentage of palmelloid cells were found under low light in both CC-4414 and CC-2344. However, unlike CC-4414, the percentage of palmelloid cells did not increase upon high light transfer in CC-2344. We believe that the development of palmelloid and cell aggregation effectively protects CC-4414 cells from stress, enabling them to survive until the stress disappears. The capacity to prolong survival under stress is what differentiates tolerant strains from sensitive strains. In fact, the palmelloid structure of *Chlamydomonas priscuii* developed at low temperatures was found to be more tolerant to high light [64]. However, given sufficient intensity and duration, the cells will likely undergo programmed cell death if the stress is prolonged.

Similar ROS management mechanisms were observed between the two strains. When these abilities are insufficient to allow cells to survive under high light, pallmeloid production and cell aggregation are required. This may be an adaptive process resulting from each strain’s specific habitat. The investigation of the molecular mechanism that leads to palmelloid production and cell aggregation is still in its infancy. Several questions remained unanswered. What are the signals that regulate the ability to generate palmelloid structures? Are all strains capable of producing palmelloid in high light? Is the ability to generate palmelloid in response to a particular stressor strain-specific? Is this characteristic shared by all high-light-tolerant isolates? Natural *Chlamydomonas* strains may hold the key to answering these questions.

## 4. Materials and Methods

### 4.1. Chlamydomonas Reinhardtii Strains and Culture Conditions

*Chlamydomonas reinhardtii* strains S1D2, CC-125, CC-407, CC-408, CC-1009, CC-1010, CC-1373, CC-1690, CC-1952, CC-2342, CC-2343, CC-2344, CC-2931, CC-2932, CC-2935, CC-2936, CC-2937, CC-2938, and CC-4414 were obtained from the Chlamydomonas Resource Center (University of Minnesota). 4A+ strain was provided by Prof. Krishna Niyogi (University of California, Berkeley). Cells were grown on agar plates or in liquid medium using sterile Tris-acetate-phosphate (TAP) medium at 25 °C and constant light at 50 µmol photons m^−2^ s^−1^ and were shook at 180 rpm. For the high light treatment, cultures were diluted to a density of 2 × 10^6^ cells mL^−1^ and were exposed to 1500 µmol photons m^−2^ s^−1^ of high light stress. Samples were collected at specified time points. To examine growth under high light stress, log-phase cells were diluted to a density of 2 × 10^6^ cells mL^−1^ and subjected to 5-fold serial dilutions. A volume of 3 microliters was spotted on TAP agar plates and exposed to the indicated light intensities for one week. For oxidative stress treatments, cells were spotted on TAP agar plates with added Rose Bengal (RB) at a final concentration of 4 µM, and hydrogen peroxide (H_2_O_2_) was added at a final concentration of 0.5 mM. Photographs were taken after 5 days.

### 4.2. Growth and Morphological Observation

To compare the growth of *Chlamydomonas* under low light and high light treatments, log-phase cultures were diluted to a density of 2 × 10^6^ cells mL^−1^ in a volume of 50 mL. Cultures were cultivated at 50 µmol photons m^−2^ s^−1^ for low light, or at 1500 µmol photons m^−2^ s^−1^ for high light. White LEDs lamps were used as a light source. Light intensity was measured using an LI-250A Light Meter (LI-COR Biosciences, Lincoln, NE, USA). Samples were taken every day for measuring the optical density at 750 nm. To examine the morphological changes, such as cell size, chlorophyll bleaching, or structural abnormalities, two hundred microliters of cultures were taken for microscopic analysis using a Canon EOS 60D microscope (Canon Inc., Tokyo, Japan). The software used for the calculation of the percentage of palmelloid was Fiji/ImageJ 2.11.0 [30,65]. Using 10 different fields of the sample observed under the microscope, the calculation employed was as follows:

% palmelloid = (palmelloid area/total cell area) × 100

### 4.3. Pigment Analysis

Cells were collected at a volume of 1 mL via centrifugation at 6000 rpm for 10 min. Pigments were extracted by vortexing the cells in 1 mL of 80% acetone until the cells were fully lysed and the pellets turned white. The absorbance of the pigment extract was measured at 470, 646, and 663 nm to calculate the total amount of chlorophyll and carotenoid using an Eppendorf BioSpectrometer (Eppendorf, Hamburg, Germany) [66]. The absorbance at 720 nm was measured to correct for turbidity [67]. The total amount of pigment content was calculated as follows:

Chl *a* (µg/mL) = 12.25 (A_663.2_ − A_720_) − 2.79 (A_646.8_ − A_720_)

Chl *b* (µg/mL) = 21.50 (A_646.8_ − A_720_) − 5.10 (A_663.2_ − A_720_)

Carotenoid (µg/mL) = [1000 (A_470_ − A_720_) − 1.82Chl *a* − 85.02Chl *b*]/198

where A_663.2_ is the absorbance at 663.2 nm, A_646.8_ is the absorbance at 646.8 nm, A_470_ is the absorbance at 470 nm, and A_720_ is the absorbance at 720 nm.

### 4.4. Lipid Peroxidation

Cultures were collected in duplicates of 10 mL via centrifugation at 5000 rpm for 10 min. One milliliter of –TBA solution consisting of 20.0% (*w*/*v*) trichloroacetic acid (TCA) and 0.01% butylated hydroxytoluene was added to one sample tube. One milliliter of +TBA solution consisting of the above plus 0.65% thiobarbituric acid (TBA) was added to the other tube. Samples were mixed and heated at 95 °C for 25 min, cooled at room temperature, and centrifuged at 12,000 rpm for 10 min. The supernatant was used for the measurement of optical density at 440, 532, and 600 nm to calculate the malondialdehyde (MDA) levels. The amount of MDA was calculated as described [68]. Malondialdehyde (MDA) level was calculated as follows:

(Abs_532_ + TBA − Abs_600_ + TBA) − (Abs_532_ − TBA − Abs_600_ − TBA) = A

(Abs_400_ + TBA − Abs_600_ + TBA) × 0.0571 = B

(A − B/157,000) × 10^6^ = MDA (nmol/mL)

where Abs_532_ is the absorbance at 532 nm, Abs_600_ is the absorbance at 600 nm, and Abs_400_ is the absorbance at 400 nm, +TBA is the solution with thiobarbituric acid (TBA), and −TBA is the solution without thiobarbituric acid (TBA).

### 4.5. Chlorophyll Fluorescence and NPQ Analysis

For photosynthesis efficiency (F_v_/F_m_) measurements, one milliliter of culture was dark acclimated for 30 min. Photosynthesis efficiency was monitored by measurement of the F_v_/F_m_ value using the Z985 Cuvette Aquapen (Qubit Systems, Kingston, ON, Canada). For nonphotochemical quenching (NPQ) measurement, four hundred microliters of culture was dark adapted for 30 min. NPQ was measured using a PAM-2500 chlorophyll fluorometer (Heinz Walz GmbH, Effeltrich, Germany). 

### 4.6. Superoxide Dismutase (SOD) Activity Assay

Cultures were collected in a volume of 5 mL and centrifuged at 8000 rpm at 4 °C for 10 min. Cells were broken via sonication in 3 mL of cold extraction buffer, consisting of 50 mM Tris-HCl pH 7.8, 1 mM EDTA, 1 mM MgCl_2_, and 1% *w*/*w* polyvinylpyrrolidone. The ability of superoxide dismutase to inhibit the photochemical reduction of nitro blue tetrazolium (NBT), which absorbs light at 560 nm, was used to determine its activity [69]. The reaction contained 50 mM potassium phosphate buffer pH 7.6, 0.1 mM EDTA, 13 mM methionine, 75 µM NBT, and 190 µL enzyme extract. Lastly, riboflavin was added, and the tubes were shaken and placed under constant light at 50 µmol photons m^−2^ s^−1^ for 10 min. The optical density at 560 nm was measured to determine the % inhibition of NBT reduction and calculated as follows:

[(OD_control_ − OD_treatment_)/OD_control_] × 100 = % inhibition of NBT reduction

% inhibition of NBT reduction = 1/50 × X as Y unit (50% inhibition = 1 unit of enzyme)

Y unit = 1/50 × % inhibition of NBT reduction

The unit of SOD was calculated by Y unit and the amount of enzyme extract as Z mL in 1 mL of total reaction mixture following the equation:

unit of SOD = (Y unit/Z mL) × 1 mL

where OD_control_ is the absorbance of the sample that was controlled, and OD_treatment_ is the absorbance of the sample that underwent treatment.

### 4.7. Proteome Analysis

#### 4.7.1. Protein Extraction

Total protein was recovered by pulverizing cells with liquid nitrogen in a mortar. Two hundred milligrams of powder samples were dissolved in 0.5% sodium dodecyl sulphate (SDS), and then vortexed continuously for 3 h at room temperature. The samples were centrifuged at 8000 rpm for 10 min at room temperature. The supernatant was transferred to a fresh 1.5 mL centrifuge tube, and subsequently, mixed with 72% trichloroacetic acid and 0.15% deoxycholate. The tube was vortexed vigorously before placing samples at −20 °C overnight. The mixture was precipitated by centrifugation at 10,000 rpm for 10 min at room temperature. The pellets were washed with cold acetone until they turned white. The pellets were resuspended in 0.5% SDS. The protein concentration was determined using the Lowry method [70].

#### 4.7.2. Protein Digestion

In-gel digestion was performed on five micrograms of protein samples. Samples were completely dissolved in 10 mM ammonium bicarbonate. The reduction of disulfide bonds was performed using 5 mM dithiothreitol in 10 mM AMBIC at 60 °C for 1 h. Sulfhydryl groups were alkylated using 15 mM Iodoacetamide in 10 mM AMBIC at room temperature for 45 min in the dark. For digestion, samples were combined with 50 ng/µL of sequencing grade trypsin (1:20 ratio) (Promega, Walldorf, Germany) and incubated overnight at 37 °C. The digested samples were dried and protonated with 0.1% formic acid prior to LC-MS/MS injection.

#### 4.7.3. Liquid Chromatography–Tandem Mass Spectrometry (LC/MS-MS)

Tryptic peptide samples were injected in triplicate (5 µL each) into an HCTUltra LC-MS system (Bruker Daltonics Ltd.; Hamburg, Germany) coupled with a nanoLC system: UltiMate 3000 LC System (Thermo Fisher Scientific; Madison, WI, USA) and electrosprayed at the flow rate of 300 nL/min to a nanocolumn (Acclaim PepMapTM 100 C18 column 50 mm internal diameter 0.075 mm). A mobile phase consisting of solvent A (0.1% formic acid) and solvent B (80% acetonitrile and 0.1% formic acid) was used to elute peptides using a linear gradient of 4–70% of solvent B at 0–20 min (the time-point of retention), followed by 90% of solvent B at 20–25 min to remove all peptides in the column. Mass spectra (MS) and MS/MS spectra were acquired in the positive ion mode throughout the range of (*m/z*) 400–1500 (Compass 1.9 software, Bruker Daltonics, Billerica, MA, USA).

#### 4.7.4. Bioinformatics and Data Analysis

DeCyder MS2.0 analysis software (GE Health-Care, Chicago, IL, USA) was used to determine the protein concentration from LC-MS data based on peptide MS signal intensities. Ion peptides were produced using the following data set: mass resolution, 0.6; typical peak width, 0.1; ion trap mass resolution, 10,000; charge status, between 1 and 4; and *m/z* shift tolerance, 0.1 u. The PepDetect module was employed to analyze the peptide. The PepMatch module was used to evaluate the signal intensity maps from each sample.

All MS/MS data from the Decyder MS analysis were acquired by applying the global variable mode of carbamidomethyl, the variable mode of oxidation (M), the peptide charge state (1+, 2+ and 3+), and a 0.1 u *m/z* tolerance. Using the Mascot software search engine version 2.3.0, these spectra were queried against UniProt databases (https://www.uniprot.org/, accessed on 10 May 2022) with Chlamydomonadales (233,134 sequences; 10 May 2022) to discover matching peptides (Matrix Science, London, UK). The identified proteins were analyzed with MultiExperiment Viewer software (MeV, version 4.9.0) and filtered via a one-way ANOVA (*p* < 0.05) [71]. BSA was used as an internal standard to normalize protein intensities from each data set. Uniport (http://www.uniprot.org/, accessed on 8 March 2023) and search tools were used to identify Gene Ontology (GO). A Venn diagram was used to determine matching proteins, similarity, and differential protein expressions. KEGG pathway databases were used to perform pathway analysis. Protein–protein interaction network was generated using STRING, version 11.5 [72]. 

### 4.8. Statistical Analysis

Data are reported as means ± standard deviation (SD). Student’s *t*-test was performed to determine statistical differences at a significant level of 0.05.

## 5. Conclusions

By investigating two *Chlamydomonas* field isolates, one of which is light-tolerant and the other of which is light-sensitive, we discovered that their initial lines of defense for ROS management were remarkably comparable. The ability to develop a palmelloid structure and a small degree of cell clustering, which shielded the cells from stress, presumably contributed to high light tolerance, as supported by proteomics data. In addition, protein degradation and autophagy were simultaneously upregulated in the tolerant strain. Hence, the light sensitivity of the sensitive strain was not attributable to an inability to reduce the amount of excess energy absorbed and eliminate ROS, but rather to a failure to produce palmelloid structure, resulting in an increase in oxidative stress and photobleaching. Likewise, under high light stress, cells must protect themselves by forming a larger structure, either in the form of a palmelloid or cluster, in order to rest till the environmental conditions are favorable. These abilities might be crucial for survival in the wild.

## Figures and Tables

**Table 1:** Proteins exclusively expressed in CC-4414 under high light with predicted function/component.

UniprotID	Function/Component	Protein Name	Peptide	*p*-Value	Fold Change
A0A7S0YFV0	Proteolysis/cell membrane	Calpain catalytic domain-containing protein	LNPKIAR	0.00	20.58
A0A7S3QLH0		PDZ domain-containing protein	VLLEALQK	0.05	15.66
A0A835WE54	SUMO transferase activity	SAP domain-containing protein	LLELFEDSYHLVSNGSVPRDMWR	0.02	20.05
A0A0S2LQ22	Photosynthesis/chloroplast thylakoid membrane	Cytochrome b6-f complex subunit 6	VVKLM	0.01	19.37
A0A250X4U9	ATP binding	Helicase ATP-binding domain-containing protein	STGGGGGGGGKGGR	0.01	19.05
A0A250X4T6	Protein phosphorylation/cell membrane	Protein kinase domain-containing protein	EGSGMIK	0.01	19.06
A0A835WI51	Nucleus	Helicase C-terminal domain-containing protein	AAARDAAAAAR	0.01	17.79
A0A835SUR5		CRC domain-containing protein	AGAKGGGAAAASGAPGGGR	0.01	17.07
A0A150G407		TFIIS N-terminal domain-containing protein	VLGGM	0.00	14.96
A0A6A0AIR0	Plant-type cell wall organization	Expansin-like EG45 domain-containing protein	GIQPG	0.01	17.35
A0A6A0AK22	Ubiquitin-protein transferase activity	RBR-type E3 ubiquitin transferase	GYVNNAIANGPASLDLRCPTPK	0.00	17.25
A0A172WYN8	mRNA processing/nucleus	Mac1	MMHSLHARVETAR	0.01	16.96
A0A2J8AGW4		E3 ubiquitin-protein ligase	GPVAAPR	0.00	16.94
A0A835SR56	Alternative oxidase activity	Ubiquinol oxidase	TMKACQDETVGQDIISR	0.01	16.93
Q6PLP9	Protein deubiquitination	Ubiquitin carboxyl-terminal hydrolase	FNLMALVGNRADIYSSR	0.00	16.90
A0A835XUN6	DNA replication/nucleus	Sld5 domain-containing protein	RGMTTFSLPEIYHER	0.00	16.87
A0A250XGZ4	RNA binding	mRNA cap-binding protein	DRYTV	0.01	16.80
A0A150G279	Protein dephosphorylation	Protein tyrosine phosphatase	FVFDSK	0.00	16.26
A0A7S3VKA6	Metalloendopeptidase activity/nucleus	SprT-like domain-containing protein	KPQPADCRGR	0.01	16.23
A0A150GXF6	Guanyl-nucleotide exchange factor activity/cytosol	SEC7 domain-containing protein	MSAEQVYHPAAEIVAIALR	0.00	15.32
A0A2K3CUA0	mRNA 5′-splice site recognition	Pre-mRNA-processing factor 39	KEEAAAEGAEGAEVK	0.00	14.64
A0A7S0VD46	Protein folding/unfolded protein binding	Prefoldin subunit 2	KPASQGVLV	0.05	13.29

**Table 2:** Proteins shared by CC-4414 and CC-2344 under high light.

UniprotID	Function/Component	Protein Name	Peptide	*p*-Value	Fold Change	
					CC-4414	CC-2344
A0A7S0YJX0	Unknown	Uncharacterized protein	PGFGGGER	0.02	18.82	17.34
A0A2J7ZZC2	Adenylate kinase activity	Adenylate kinase	EGMTVVTR	0.01	16.60	19.50
A0A2K3DSM2	Potassium:proton antiporter activity/plasma membrane	Na_H_Exchanger domain-containing protein	GVGGMGVGMGGGAAGTGPGGGGGGGGR	0.00	15.63	17.68
A8JFC7	Unknown	Copper homeostasis protein cutC homolog	VELCAALIEGGITPSAGMIR	0.01	15.61	19.46

**Table 3:** Proteins exclusively expressed in CC-2344 under high light.

UniprotID	Function/Component	Protein Name	Peptide	*p*-Value	Fold Change
A0A835XQZ1	Membrane	Basic proline-rich protein-like	ASLGVLLSSGAGGGGGGKGAGAK	0.01	26.81
A0A835SW25	ATP binding/membrane	Protein kinase domain-containing protein	SSSNR	0.01	20.93
A0A2K3CS73	Unknown	DUF4604 domain-containing protein	GGLRTAQGGGGAGK	0.04	20.31
A0A2J8A5D8	Nucleic acid binding	CCHC-type domain-containing protein	VLLPPSK	0.01	19.93
D8U4G9	Unknown	ANK_REP_REGION domain-containing protein	VPGLAGALKR	0.01	19.75
A0A250X3Y5	Unknown	DUF4378 domain-containing protein	MSGGG	0.00	19.72
A0A835Y1G5	Unknown	Protein kinase domain-containing protein	AATLPRAAAPNAAAK	0.00	19.14
A0A250XQV9	Unknown	Uncharacterized protein	SSTATR	0.01	18.16
D8UA57	Unknown	DUF4485 domain-containing protein	DAIGSQRGAK	0.01	18.03
A0A835WLJ1	Sphingomyelin phosphodiesterase activity	ANK_REP_REGION domain-containing protein	AAGPGAGAGADVAR	0.00	17.95
A0A2K3DJS2	Unknown	CobW C-terminal domain-containing protein	APAPAPGPASGPAPAPGPASGSAGMAAMCAEERR	0.00	17.77
Q8WKX7	Chloroplast/ endonuclease activity	LAGLIDADG_2 domain-containing protein	MIIKSGEK	0.01	17.50
A0A1W5IVS0	Unknown	RlsD	GSHDGVGR	0.00	17.30
A0A835T9S6	Phosphorus-oxygen lyase activity	Guanylate cyclase domain-containing protein	RTGAPVQQHR	0.01	16.89
A0A2K3CW68	Guanylate cyclase activity/plasma membrane	Guanylate cyclase domain-containing protein	TRIQPVLR	0.01	16.20
A0A2K3CPC2	Unknown	GSCFA domain-containing protein	DPGLPSECVGR	0.04	15.88

## Data Availability

Mass spectrometry proteomics data are available via ProteomeXchange: PXD040080 and JPOST: JPST002038.

## Supplementary Materials

The supporting information can be downloaded at: https://www.mdpi.com/article/10.3390/ijms24098374/s1.
