# Supplementary material for: Palmelloid Formation and Cell Aggregation Are Essential Mechanisms for High Light Tolerance in a Natural Strain of Chlamydomonas reinhardtii
Source: Int J Mol Sci. 2023 May 6;24(9):8374. doi: 10.3390/ijms24098374 (PMC10179368; doi:10.3390/ijms24098374)
Supplement: Supplementary file 1 [file ijms-24-08374-s001.zip › ijms-2369728-supplementary.pdf]

International Journal of
Molecular Sciences

MDPI

Article

Palmelloid Formation and Cell Aggregation Are Essential Mechanisms for High Light Tolerance in a Natural Strain of *Chlamydomonas reinhardtii*

Nittaya Suwannachuen [1,2], Kantinan Leetanasaksakul [3], Sittiruk Roytrakul [3], Narumon Phaonakrop [3], Siriwan Thaisakun [3], Peerapat Roongsattham [1,2], Chatchawan Jantasuriyarat [1,2], Nuttha Sanevas [4] and Anchalee Sirikhachornkit [1,2,*]

Table S1. Proteins with predicted domain/function under biological process.

Biological process	Protein name	Peptide	P-value	Fold change
**Organic substance metabolic process**				
Proteolysis	Calpain catalytic domain-containing protein	LNPKIAR	0.00	20.58
	PDZ domain-containing protein	VLLEALQK	0.05	15.66
Dolichol-linked oligosaccharide biosynthetic process	UDP-N-acetylglucosamine transferase subunit ALG14	KPATARTKPAK	0.00	19.88
Nuclear-transcribed mRNA catabolic process	PHD-type domain-containing protein	AAAAAAEAGAGGKK	0.02	19.07
Methylation	2-methoxy-6-polyprenyl-1,4-benzoquinol methylase	MALRSSTGFR	0.01	19.00
Histone H3-K9 demethylation	JmjC domain-containing protein	VLDSSALR	0.02	17.93
Cysteinyl-tRNA aminoacylation	cysteine--tRNA ligase	VAEMEAMVAK	0.00	17.86
Protein glycosylation	EGF-like domain-containing protein	MFLNDWAVR	0.01	17.82
N-terminal peptidyl-methionine acetylation	SET domain-containing protein	SVPADPASYVVR	0.05	17.56
Peptidyl-tyrosine sulfation	protein-tyrosine sulfotransferase	DVDGSLSSR	0.01	17.12
mRNA processing	RRM domain-containing protein	EADRLAAER	0.05	23.32
	Mac1	MMHSLHARVETAR	0.01	16.96
	E3 ubiquitin-protein ligase	GPVAAPR	0.00	16.94
DNA replication	Sld5 domain-containing protein	RGMTTFSLPEIYHER	0.00	16.87
Oligosaccharide metabolic process	Mannosyl-oligosaccharide glucosidase	SLAASASLYKK	0.01	14.87
mRNA 5'-splice site recognition	Pre-mRNA-processing factor 39	KEEAAAEGAEGAEVK	0.00	14.64
tRNA splicing, via endonucleolytic cleavage and ligation	tRNA_lig_CPD domain-containing protein	AAPRDAGAGAEPMAVDGGGSCAK	0.00	14.59
**Photosynthesis**	Cytochrome b6-f complex subunit 6	VVKLM	0.01	19.37
**Protein modification process**				
Protein phosphorylation	Protein kinase domain-containing protein	EGSGMIK	0.01	19.06
	Protein kinase domain-containing protein	LWHSLDASQLLKPAPSVVARNR	0.02	19.05
	Protein kinase domain-containing protein	SLAQACWNADPRK	0.00	18.33
	Protein kinase domain-containing protein	LTEGPK	0.01	18.09
	Protein kinase domain-containing protein	KPAIVHR	0.01	18.04
	Protein kinase domain-containing protein	NETNPTPWPILLFR	0.00	17.91
	Alpha-protein kinase vwkA	DPMMTR	0.01	17.70
	Protein kinase domain-containing protein	VDRMIYAMK	0.01	17.53
	Protein kinase domain-containing protein	GPAASAGAHVGFQSGGAVAGGGAASMGSGMR	0.01	16.78
Protein deubiquitination	Ubiquitin carboxyl-terminal hydrolase	FNLMALVGNRADIYSSR	0.00	16.90
Protein dephosphorylation	Protein-tyrosine-phosphatase	FVFDSK	0.00	16.26
**Cellular component organization or biogenesis**				
Ribosome biogenesis	60S ribosomal protein L7a	GATFLSVAGIAQMAPK	0.00	19.03

Cell projection organization	RING-type domain-containing protein	LGTSNRTDAVAASQPPPLDPFTVK	0.00	18.63
Plant-type cell wall organization	Expansin-like EG45 domain-containing protein	GIQPG	0.01	17.35
**Transcription**				
DNA-templated transcription	DNA-directed RNA polymerase subunit	SIHNPIYAVSYR	0.02	18.45
	DNA-directed RNA polymerase subunit beta	TVTLPPDSSGAVQR	0.01	18.30
	DNA-directed RNA polymerase subunit beta	SFSTTK	0.00	17.32
Regulation of DNA-templated transcription	PAS domain-containing protein	ASYMTGLIVGMPAAAMVK	0.01	17.02
	PAS domain-containing protein	VIDSVR	0.00	16.48
DNA-templated transcription termination	SAM domain-containing protein	GVAKVSAVADR	0.00	15.68
Transcription by RNA polymerase III	General transcription factor 3C polypeptide 3	ALAQFALVAK	0.00	13.44
**Cellular metabolic process**				
Cyclic nucleotide biosynthetic process	Guanylate cyclase domain-containing protein	VRMGMHAGISNEADVAYNK	0.01	18.18
	Guanylate cyclase domain-containing protein	YPPGMLPPGISLTTDR	0.00	18.04
	Guanylate cyclase domain-containing protein	AVGPGSYELMVR	0.01	17.98
	Guanylate cyclase domain-containing protein	ASTATAGAAAGAAGGSR	0.04	17.49
Adenine salvage	Adenine phosphoribosyltransferase	AAFVPLRKPGK	0.00	17.96
Phosphorylation	PPDK_N domain-containing protein	VDYMDDR	0.00	15.85
de novo' pyrimidine nucleobase biosynthetic process	Uridine 5'-monophosphate synthase	TFIAENQTK	0.00	14.78
Protein folding	Prefoldin subunit 2	KPASQGVLV	0.05	13.29

**Biological process**	**Protein name**	**Peptide**	**P-value**	**Fold change**
**Translation**				
Formation of cytoplasmic translation initiation complex	Eukaryotic translation initiation factor 3 subunit E (eIF3e)	LQADVDPIIK	0.01	18.06
Translation	30S ribosomal protein S18, chloroplastic	NIKPIMPPK	0.01	17.39
	Plastid ribosomal protein S20	QSTMLR	0.00	17.24
**Cell cycle**	Kinetochore protein SPC25	MSIDIK	0.01	18.04
**Cellular response to stress**				
Cellular response to DNA damage stimulus	Peptidase HMCES	SATDRASHTIDQVSGR	0.00	17.58
	SprT-like domain-containing protein	KPQPADCRGR	0.01	16.23
**Signal transduction**	RING-type domain-containing protein	RSAAQGSGGPSQGPQPGDGQGGK	0.00	16.51
**Other**				
Cilium assembly	Apple domain-containing protein	NLLGPAAR	0.00	17.59
Intracellular protein transport	Adaptin_N domain-containing protein	SDNMG	0.01	17.35
Protein transport	SEC7 domain-containing protein	MSAEQVYHPAAEIVAIALR	0.00	15.32

Table S2. Proteins with predicted domain/function under cellular component.

Cellular component	Protein name	Peptide	P-value	Fold change
**Membrane**				
Cell membrane	Calpain catalytic domain-containing protein	LNPKIAR	0.00	20.58
	Beta_helix domain-containing protein	QSINGSQGDSSGDMGGER	0.01	18.93
	SPX domain-containing protein	TGSLRHVDTK	0.00	18.61
	Uncharacterized protein	AAEDPR	0.00	18.59
	Peroxisomal membrane protein PMP22	MLLARLPPVPR	0.01	18.52
	Transmembrane protein	TAHILPGVARELMLR	0.00	18.48
	N-acetyltransferase domain-containing protein	LGGMALMLLGAGVAAAACR	0.01	18.32
	Ig-like domain-containing protein	AGVISK	0.01	18.28
	RING-type domain-containing protein	NTSGRSR	0.00	18.22
	RING-CH-type domain-containing protein	ARVVVGFGGLVR	0.00	18.17
	TRP domain-containing protein	VLNSCVTGGRTLK	0.01	18.15
	Guanylate cyclase domain-containing protein	YPPGMLPPGISLTTDR	0.00	18.04
	Thioredoxin domain-containing protein	SAAVTR	0.00	17.84
	ORC_WH_C domain-containing protein	LERGDGAAAAAVQPPR	0.00	17.83
	Protein kinase domain-containing protein	VYHTWGIAAGAAGPLR	0.01	17.66
	Uncharacterized protein	PLGALLNSTMR	0.01	17.44
	Protein-tyrosine sulfotransferase	DVDGSLSSR	0.01	17.12
	PAS domain-containing protein	ASYMTGLIVGMPAAAMVK	0.01	17.02
	Protein kinase domain-containing protein	EVAFAFAELALK	0.01	16.80
	DUF3707 domain-containing protein	LTAAARSQAR	0.00	16.75
	FAD_binding_3 domain-containing protein	MTSMAPAGLGQR	0.03	16.56
	PAS domain-containing protein	VIDSVR	0.00	16.48
	RGS domain-containing protein	TGFDLMVSVKLFLGR	0.03	16.39
	RING-type domain-containing protein	RGDMAMCK	0.00	15.56
	C-type lectin domain-containing protein	EGELQPQGAGGLGGQSR	0.00	15.51
	PKD_channel domain-containing protein	NAAELALR	0.00	14.79
	RING-type domain-containing protein	VQLLAGPLTWLLVMAAYNAVGR	0.00	13.91
Extrinsic component of mitochondrial inner membrane	2-methoxy-6-polyprenyl-1,4-benzoquinol methylase	MALRSSTGFR	0.01	19.00
Plasma membrane	EF-hand domain-containing protein	IHALGFRK	0.00	17.86
**Cell projection**	SPX domain-containing protein	GGESG	0.02	20.93
**Intracellular**				
Cytoplasm	ASH domain-containing protein	LVGVATNPEK	0.01	16.15

	RZ-type domain-containing protein	AARVVGMTTSGVAR	0.02	20.57
	Adenine phosphoribosyltransferase	AAFVPLRKPGK	0.00	17.96
	Amidohydro-rel domain-containing protein	AAAFVMSPPLRSAEHAPALR	0.01	17.15
Chloroplast thylakoid membrane	Cytochrome b6-f complex subunit 6	VVKLM	0.01	19.37
Cytosol	SEC7 domain-containing protein	MSAEQVYHPAAEIVAIALR	0.00	15.32
**Organelle**				
Endoplasmic reticulum	UDP-N-acetylglucosamine transferase subunit ALG14	KPATARTKPAK	0.00	19.88
Cilium	RING-type domain-containing protein	LGTSNRTDAVAASQPPPLDPFTVK	0.00	18.63
Chloroplast	DNA-directed RNA polymerase subunit beta	TVTLPPDSSGAVQR	0.01	18.30
	Hypothetical chloroplast protein RF1	SSNLNTKDNSSNK	0.01	17.64
	30S ribosomal protein S18	NIKPIMPPK	0.01	17.39
	DNA-directed RNA polymerase subunit beta	SFSTTK	0.00	17.32
	Mac1	MMHSLHARVETAR	0.01	16.96
	TPR_REGION domain-containing protein	WGLMVASCYR	0.01	16.56
	General transcription factor 3C polypeptide 3	ALAQFALVAK	0.00	13.44
	Elongation factor Tu	ITMVVELINPIAIEK	0.00	12.37
**Cellular component**	**Protein name**	**Peptide**	**P-value**	**Fold change**
Peroxisome	Peroxisome biogenesis factor 10	GGHGR	0.01	18.22
Ndc8 complex	Kinetochore protein SPC25	MSIDIK	0.01	18.04
Nucleus	TFIIS N-terminal domain-containing protein	VLGGM	0.00	14.96
	JmjC domain-containing protein	VLDSSALR	0.02	17.93
	Helicase C-terminal domain-containing protein	AAARDAAAAAR	0.01	17.79
	SBP-type domain-containing protein	LVRAAEALGR	0.00	17.29
	CRC domain-containing protein	AGAKGGGAAAASGAPGGGR	0.01	17.07
	E3 ubiquitin-protein ligase	GPVAAPR	0.00	16.94
	Sld5 domain-containing protein	RGMTTFSLPEIYHER	0.00	16.87
	RING-type domain-containing protein	RSAAQGSGGPSQGPQPGDGQGGK	0.00	16.51
	SprT-like domain-containing protein	KPQPADCRGR	0.01	16.23
Golgi apparatus	EGF-like domain-containing protein	MFLNDWAVR	0.01	17.82
MKS complex	Apple domain-containing protein	NLLGPAAR	0.00	17.59
Intracellular organelle	Ubiquinol oxidase	TMKACQDETVGQDIISR	0.01	16.93
Nucleosome	Histone H4	AELLEXK	0.01	16.25
**Ribonucleoprotein complex**				
Cytosolic large ribosomal subunit	60S ribosomal protein L7a	GATFLSVAGIAQMAPK	0.00	19.03
Eukaryotic 43S preinitiation complex	Eukaryotic translation initiation factor 3 subunit E	LQADVDPIIK	0.01	18.06

Small ribosomal subunit	Plastid ribosomal protein S20	QSTMLR	0.00	17.24
Commitment complex	Pre-mRNA-processing factor 39	KEEAAAEGAEGAEVK	0.00	14.64
**Organelle membrane**				
Endoplasmic reticulum membrane	MSP domain-containing protein	VQVTMK	0.00	18.65
	Mannosyl-oligosaccharide glucosidase	SLAASASLYKK	0.01	14.87
**Protein complex**				
DNA-directed RNA polymerase complex	DNA-directed RNA polymerase subunit	SIHNPIYAVSYR	0.019	18.455
Membrane coat	Adaptin_N domain-containing protein	SDNMG	0.006	17.345
NatC complex	SET domain-containing protein	MDSGATR	0.007	17.304
Exon-exon junction complex	RRM domain-containing protein	APQQASGLGNGAPSGGGASGGSGSR	0.009	17.068
Prefoldin complex	Prefoldin subunit 2	KPASQGVLV	0.045	13.288
**Other**				
Nuclear pore	Nucleoporin Nup186/Nup192/Nup205	ASLDGALSALATIKDLAPR	0.01	18.00
Extracellular region	Chitin-binding type-2 domain-containing protein	TGTVMGFGLQVPAILGSATVNAAMLLMDANK	0.00	17.22
Chloroplast membrane	MYND-type domain-containing protein	LALASAR	0.01	17.13

Table S3. Proteins with predicted domain/function under molecular function.

Molecular function	Protein name	Peptide	P-value	Fold change
**Catalytic activity**				
Calcium-dependent cysteine-type endopeptidase activity	Calpain catalytic domain-containing protein	LNPKIAR	0.00	20.58
5'-3' DNA helicase activity	RZ-type domain-containing protein	AARVVGMTTSGVAR	0.02	20.57
Peptide-methionine (S)-S-oxide reductase activity	peptide-methionine (S)-S-oxide reductase	MATAYAVALRSASSR	0.00	20.41
SUMO transferase activity	SAP domain-containing protein	LLELFEDSYHLVSNGSVPRDMWR	0.02	20.05
Sphingomyelin phosphodiesterase activity	Ankyrin repeat domain-containing protein	NGDQAMLR	0.01	19.12
	ANK_REP_REGION domain-containing protein	GHGEACR	0.00	19.11
2-octaprenyl-6-methoxy-1,4-benzoquinone methylase activity	2-methoxy-6-polyprenyl-1,4-benzoquinol methylase	MALRSSTGFR	0.01	19.00
Acetyltransferase activity	N-acetyltransferase domain-containing protein	LGGMALMLLGAGVAAAACR	0.01	18.32
Phosphorus-oxygen lyase activity	Guanylate cyclase domain-containing protein	AVGPGSYELMVR	0.01	17.98
	Guanylate cyclase domain-containing protein	ASTATAGAAAGAAGGSR	0.04	17.49
Adenine phosphoribosyltransferase activity	Adenine phosphoribosyltransferase	AAFVPLRKPGK	0.00	17.96
Histone H3K9 demethylase activity	JmjC domain-containing protein	VLDSSALR	0.02	17.93
Glycosyltransferase activity	EGF-like domain-containing protein	MFLNDWAVR	0.01	17.82
Peptidase activity	Peptidase HMCES	SATDRASHTIDQVSGR	0.00	17.58
Catalytic activity	Thioredoxin domain-containing protein	DMVLRFFNASPAEYQVVFTK	0.00	17.53
	MOSC domain-containing protein	LPAPLPMNR	0.00	14.45

Hydrolase activity	M20_dimer domain-containing protein	AFSDDIMR	0.00	17.50
	Amidohydro-rel domain-containing protein	AAAFVMSPPLRSAEHAPALR	0.01	17.15
Ubiquitin-protein transferase activity	RBR-type E3 ubiquitin transferase	GYVNNAIANGPASLDLRCPTPK	0.00	17.25
Protein-tyrosine sulfotransferase activity	protein-tyrosine sulfotransferase	DVDGSLSSR	0.01	17.12
Alternative oxidase activity	Ubiquinol oxidase	TMKACQDETVGQDIISR	0.01	16.93
Cysteine-type deubiquitinase activity	Ubiquitin carboxyl-terminal hydrolase	FNLMALVGNRADIYSSR	0.00	16.90
Protein tyrosine/serine/threonine phosphatase activity	Protein-tyrosine-phosphatase	FVFDSK	0.00	16.26
Metalloendopeptidase activity	SprT-like domain-containing protein	KPQPADCRGR	0.01	16.23
Serine-type peptidase activity	PDZ domain-containing protein	VLLEALQK	0.05	15.66
Transferase activity	RING-type domain-containing protein	RGDMAMCK	0.00	15.56
Glc3Man9GlcNAc2 oligosaccharide glucosidase activity	Mannosyl-oligosaccharide glucosidase	SLAASASLYKK	0.01	14.87
Orotate phosphoribosyltransferase activity	Uridine 5'-monophosphate synthase	TFIAENQTK	0.00	14.78
RNA ligase (ATP) activity	tRNA_lig_CPD domain-containing protein	AAPRDAGAGAEPMAVDGGGSCAK	0.00	14.59
**Nucleic acid binding**				
RNA binding	RRM domain-containing protein	EADRLAAER	0.05	23.32
	PHD-type domain-containing protein	AAAAAAEAGAGGKK	0.02	19.07
	60S ribosomal protein L7a	GATFLSVAGIAQMAPK	0.00	19.03
	RRM domain-containing protein	APQQASGLGNGAPSGGGASGGSGSR	0.01	17.07
	mRNA cap-binding protein	DRYTV	0.01	16.80
	HTH La-type RNA-binding domain-containing protein	GFGGRAR	0.00	14.77
Translation initiation factor activity	Eukaryotic translation initiation factor 3 subunit E	LQADVDPIIK	0.01	18.06
rRNA binding	30S ribosomal protein S18, chloroplastic	NIKPIMPPK	0.01	17.39
DNA binding	DNA-directed RNA polymerase subunit	SIHNPIYAVSYR	0.02	18.45
	DNA-directed RNA polymerase subunit beta	TVTLPPDSSGAVQR	0.01	18.30
	DNA-directed RNA polymerase subunit beta	SFSTTK	0.00	17.32
	SBP-type domain-containing protein	LVRAAEALGR	0.00	17.29
	RING-type domain-containing protein	RSAAQGSGGPSQGPQPGDGQGGK	0.00	16.51
	Histone H4	AELLEXK	0.01	16.25
Small ribosomal subunit rRNA binding	Plastid ribosomal protein S20	QSTMLR	0.00	17.24
mRNA binding	Mac1	A0A172WYN8	0.01	16.96
Nucleic acid binding	E3 ubiquitin-protein ligase	GPVAAPR	0.00	16.94
	Exonuclease domain-containing protein	KMACHNGAAFGTQGGK	0.01	16.55
Double-stranded DNA binding	SAM domain-containing protein	GVAKVSAVADR	0.00	15.68

**Molecular function**	**Protein name**	**Peptide**	**P-value**	**Fold change**
**Electron transporter**	Cytochrome b6-f complex subunit 6	VVKLM	0.01	19.37

**ATP binding**	Protein kinase domain-containing protein	EGSGMIK	0.01	19.06
	Protein kinase domain-containing protein	LWHSLDASQLLKPAPSVVARNR	0.02	19.05
	Helicase ATP-binding domain-containing protein	STGGGGGGGGKGGR	0.01	19.05
	Protein kinase domain-containing protein	SLAQACWNADPRK	0.00	18.33
	SNF2 family Chromodomain-helicase protein	MWAAGKHAVLADDMGLGK	0.02	18.24
	Protein kinase domain-containing protein	LTEGPK	0.01	18.09
	Protein kinase domain-containing protein	KPAIVHR	0.01	18.04
	Protein kinase domain-containing protein	NETNPTPWPILLFR	0.00	17.91
	cysteine--tRNA ligase	VAEMEAMVAK	0.00	17.86
	Helicase ATP-binding domain-containing protein	GSRVQSSSSEPGR	0.01	17.86
	Helicase C-terminal domain-containing protein	AAARDAAAAAR	0.01	17.79
	Alpha-protein kinase vwkA	DPMMTR	0.01	17.70
	Protein kinase domain-containing protein	VDRMIYAMK	0.01	17.53
	Protein kinase domain-containing protein	GPAASAGAHVGFQSGGAVAGGGAASMGSGMR	0.01	16.78
	PPDK_N domain-containing protein	VDYMDDR	0.00	15.85
	P-loop containing nucleoside triphosphate hydrolase protein	GAARFVLLEER	0.03	11.18
**Kinase activity**	Non-specific serine/threonine protein kinase	CVLAYVR	0.01	18.81
	Non-specific serine/threonine protein kinase	EVANMGPQGNAASRR	0.00	17.81
	FAT domain-containing protein	TGHEVKGR	0.00	17.52
	MYND-type domain-containing protein	LALASAR	0.01	17.13
**Ion binding**				
Metal ion binding	C3H1-type domain-containing protein	ANNDCPR	0.00	18.41
	Peroxisome biogenesis factor 10	GGHGR	0.01	18.22
	Fe-ADH domain-containing protein	APLVSDLR	0.01	17.08
Zinc ion binding	RING-CH-type domain-containing protein	ARVVVGFGGLVR	0.00	18.17
Calcium ion binding	EF-hand domain-containing protein	GDEGAIQAMAR	0.02	16.59
	Calmodulin	LVFTVVGASVK	0.00	16.25
Photoreceptor activity	PAS domain-containing protein	ASYMTGLIVGMPAAAMVK	0.01	17.02
	PAS domain-containing protein	VIDSVR	0.00	16.48

**protein binding**				
RNA polymerase binding	WW domain-containing protein	ENISVIMIMSSRGVDNLAR	0.01	15.33
Unfolded protein binding	Prefoldin subunit 2	KPASQGVLV	0.05	13.29
**Other**				
Malate transmembrane transporter activity	SPX domain-containing protein	TGSLRHVDTK	0.00	18.61
Cation channel activity	EF-hand domain-containing protein	IHALGFRK	0.00	17.86
Chitin binding	Chitin-binding type-2 domain-containing protein	TGTVMGFGLQVPAILGSATVNAAMLLMDANK	0.00	17.22
Scavenger receptor activity	C-type lectin domain-containing protein	EGELQPQGAGGLGGQSR	0.00	15.51
Guanyl-nucleotide exchange factor activity	SEC7 domain-containing protein	MSAEQVYHPAAEIVAIALR	0.00	15.32
GTP binding	Elongation factor Tu	ITMVVELINPIAIEK	0.00	12.37
